# A Manual of Hypodermatic Medication

**Published:** 1892-05

**Authors:** 


A Manual of Hypodermatic Medication: The Treatment of Diseases by the Hypodermic or Subcutaneous Method. By Roberts Bartholow, A. M., M. D., LL. D., Emeritus Professor of Materia Medica, General Therapeutics and Hygiene in the Jefferson College of Philadelphia; Honorary Fellow of the Royal Society of Edinburgh; Honorary Member of the Société Medico-Practique de Paris; Fellow of the College of Physicians of Philadelphia; Member of the American Philosophical Society; Fellow of the Medico-Chirurgical Faculty of Maryland; Honorary Member of the various State and local Societies; Author of a Treatise on the Practice of Medicine; of a Treatise on Materia Medica and Therapeutics; of a Manual of Medical Electricity, etc., etc. Fifth edition, revised and enlarged. Philadelphia: J. B. Lippincott Company. London: 0 Henrietta street, Covent Garden. 1891.

It seems like meeting an old friend who has been absent for some time and come again to visit us, to once more find this valuable book on Hypodermatic Medication, by Bartholow, on our library table. The first book that we ever saw on this subject was written by this author, and he has continued to be recognized as almost alone in this field of authorship. When his first edition was published, its range was limited to a few drugs only, and the work was necessarily much smaller than the present treatise. Now we have a formidable array of drugs that can be administered hypodermatically, and their physiological effects are oftentimes so different and so much enhanced by this method over the ordinary stomachal administration, that we must needs have a separate and distinct law laid down to guide us in this field.

The present treatise, although professing to be the fifth edition of a former issue, is essentially a new book. Most of the articles have been either recast or rewritten, and so much new matter has been introduced that it is no longer the same book. It is something like the boy's jack-knife with new blades, new handle, new back, and new rivets, but has all been done by the same master, who thoroughly understands the old as well as the new, and has maintained the usefulness of the instrument by carefully forging and tempering its new parts. Every practitioner of medicine, no matter what his specialty, should possess Bartholow's manual, for it will not only serve as a guide to him in doubtful conditions, but will instruct him in many hitherto unknown ways. We are pleased to see that the author has retained the word hypodermatic, which he introduced in the fourth edition, in place of the old familiar term hypodermic, which was employed in the earlier issues. This book is a handsomely printed small octavo of 540 pages, including an excellent index, and is easily worth the price which the publishers mark it at.

